# A knowledge discovery methodology from EEG data for cyclic alternating pattern detection

**DOI:** 10.1186/s12938-018-0616-z

**Published:** 2018-12-18

**Authors:** Fátima Machado, Francisco Sales, Clara Santos, António Dourado, C. A. Teixeira

**Affiliations:** 1 CISUC-Centro de Informática e Sistemas da Universidade de Coimbra, Departamento de Engenharia Informática, Faculdade de Ciências e Tecnologia, Universidade de Coimbra, 3030-290 Coimbra, Portugal; 2 Centro Integrado de Epilepsia, Centro Hospitalar e Universitário de Coimbra, Coimbra, Portugal; 3 Centro de Medicina do Sono do Hospital Geral Coimbra, Coimbra, Portugal

**Keywords:** Cyclic alternating pattern, A-phase detection, EEG processing, Knowledge discovery in data

## Abstract

**Background:**

Detection and quantification of cyclic alternating patterns (CAP) components has the potential to serve as a disease bio-marker. Few methods exist to discriminate all the different CAP components, they do not present appropriate sensitivities, and often they are evaluated based on accuracy (AC) that is not an appropriate measure for imbalanced datasets.

**Methods:**

We describe a knowledge discovery methodology in data (KDD) aiming the development of automatic CAP scoring approaches. Automatic CAP scoring was faced from two perspectives: the binary distinction between A-phases and B-phases, and also for multi-class classification of the different CAP components. The most important KDD stages are: extraction of 55 features, feature ranking/transformation, and classification. Classification is performed by (i) support vector machine (SVM), (ii) k-nearest neighbors (k-NN), and (iii) discriminant analysis. We report the weighted accuracy (WAC) that accounts for class imbalance.

**Results:**

The study includes 30 subjects from the CAP Sleep Database of Physionet. The best alternative for the discrimination of the different A-phase subtypes involved feature ranking by the minimum redundancy maximum relevance algorithm (mRMR) and classification by SVM, with a WAC of 51%. Concerning the binary discrimination between A-phases and B-phases, k-NN with mRMR ranking achieved the best WAC of 80%.

**Conclusions:**

We describe a KDD that, to the best of our knowledge, was for the first time applied to CAP scoring. In particular, the fully discrimination of the three different A-phases subtypes is a new perspective, since past works tried multi-class approaches but based on grouping of different sub-types. We also considered the weighted accuracy, in addition to simple accuracy, resulting in a more trustworthy performance assessment. Globally, better subtype sensitivities than other published approaches were achieved.

## Background

A cyclic alternating pattern (CAP) sequence is composed by a succession of CAP cycles, each one composed by two types of phases: The A-phases and the B-phases. The A-phases in lighter stages of comas are closely related to hyperventilation, restless, increase of pulse rate, and can be associated with increase in muscle activity. In contrast, autonomic and muscular activities are attenuated during the B-phases. Investigations discovered also that CAP is a physiologic component of non-rapid eye movement (NREM) sleep stages and do not occur, under normal conditions, in rapid eye movement (REM) [1, 2]. CAP sequences tend to appear associated with some dynamic sleep events like a change in sleep stages, falling asleep, or arousal without awaking [3]. However, some pathological conditions generate CAP sequences in REM, therefore it has potential to be used as a prognostic element of such diseases. Higher CAP rates are present in some types of insomniac patients [4], epilepsies [5], among other disorders. Thus, CAP quantification could be used as diseases biomarker, for example for seizure prediction [6].

The CAP definition and scoring rules are described in [7]. CAP is defined there as “a periodic EEG activity of NREM sleep characterized by sequences of transient electrocortical events, that are distinct from the background electroencephalogram (EEG) activity and reoccur at up to 1-minute intervals”. An A-phase is considered as a transient phenomenon translated by an increase in amplitude and/or frequency which is clearly distinguishable from background activity, i.e. from the B-phase, and is associated to a brain activation, including cortical arousal. For this reason, an A-phase is a potential trigger of somatomotor activities. Each phase can have a duration between 2 and 60 s. The mean duration of CAP sequences in young adults is two and half minutes, which contains in average six CAP cycles [1]. If an A-phase is not preceded and succeeded by another A-phase in 60 s, it is considered an isolated A-phase and defined to not belonging to a CAP cycle. To form a CAP sequence, at least two consecutive CAP cycles are required. An A-phase can be composed by high-voltage slow waves which are manifestations of EEG synchrony, by low-amplitude fast rhythms that are evidence of the EEG desynchrony and be associated with typical waveforms (for example K-complexes). Depending on the associated waveforms and on the percentage of each wave type, a given A-phase is classified into three different subtypes:

- Subtype A1: It is composed by high-voltage slow waves. This subtype can be classified by an increase in amplitude of at least a third of the normal background activity. The synchronized EEG pattern must occupy more than 80% of the epoch to be classified as A1. The associated waveforms with this subtype are bursts, and K-complex sequences.
- Subtype A2: This subtype has elements from subtypes A1 and A3, and, therefore, it is composed by a mixture of fast and slow rhythms. The elements from the subtype A1 must occupy between 50 and 80% of the length of an entire A-phase. In this subtype, the typical waveforms are polyphasic bursts.
- Subtype A3: Rapid low voltage rhythms prevail in this subtype, and there is an increase in frequency compared to the background. K-alpha, EEG arousals, and polyphasic bursts are the EEG waveforms associated with this subtype.

To mention also that the A-phases subtypes present different signatures in the conventional EEG frequency bands: delta ($\delta = [1,4]$ Hz), theta (*θ *= [4, 8] Hz), alpha (*α *= [8, 13] Hz), sigma (*σ *= [13, 16] Hz) and beta (*β *= [16, 35] Hz) [8].

Different algorithms have been proposed in the last years for automatic scoring of A-phases and B-phases. Barcaro et al. proposed a detection method for A-phases [9–11], more precisely in [10, 11] these authors considered only three classes: B, A1 and A2/A3 phases. The association of subtypes A2 and A3 was due to their similarity, as reported in the literature. The rationale of the methodology was the same in both papers [10, 11]: an amplitude feature was computed for each of the conventional frequency bands and then compared with a threshold. When one of the five features crossed the threshold a tentative A-phase detection was performed. The type assigned to the A-phases detected (A1 or A2/A3) depended on which features crossed the threshold. The best reported results were the ones proposed with the methodology presented in [11], which had a correctness of 83.5% for the binary discrimination between A-phases and B-phases, and 73.7% for the multi-class distinction between A1, A2/A3 subtypes.

A model based on feedback loops that simulates the EEG activity was proposed by [12]. This methodology was used to detect K-complexes and vertex waves and detected CAP sequences by considering four rhythm generators, which corresponded to different frequency bands (*δ*, *α*, *θ* and *σ*). The results obtained with this algorithm pointed for a mean sensitivity of 90% [12].

A method based on wavelets and genetic algorithm was proposed in [13] to identify the A-phases independently of the subtype. Basically, the signal was decomposed into five signals corresponding to the different frequency bands, using the discrete wavelet transform. A feature based on amplitude was computed for each decomposed signal. The A-phases were detected comparing the features to a threshold. The accuracy reported was 79%.

Stam and van Dijk [14] published a study that defined the synchronization likelihood (SL) between two EEG channels and proposed a method to calculate this value. This measurement was applied for A1 detection [15], where the signal was filtered between 0.25 and 2.5 Hz. They concluded that during sleep the levels of SL in this frequency range had significant fluctuations with CAP occurrence. This feature presented a good performance in distinguishing the A1 subtype from the background [16]. Although this was only true for NREM stage 2. For the others sleep stages this feature cannot distinguish A1 or the other subtypes [15, 16].

In 2012, a method using different classifiers was proposed for the binary discrimination between A-phases and B-phases, using seven EEG features. The classifiers considered were support vector machine (SVM), linear discriminant analysis (LDA), Adaboost, and artificial neural networks (ANN) [17]. Five out of seven features were related with the signal amplitude filtered in the conventional frequency bands. The other two features were the *Hjorth activity* and EEG variance. The reported accuracy for LDA, SVM, Adaboost, and ANN were 84.9 ± 4.9%, 81.9 ± 7.8%, 79.4 ± 5.5% and 81.5 ± 6.4%, respectively. Concerning the sensitivity, the results for LDA, SVM, Adaboost, and ANN were 72.5 ± 10.9%, 70.1 ± 8.6%, 68.5 ± 6.7% and 72.9 ± 7.5%, respectively. Finally, the specificity for LDA, SVM, Adaboost, and ANN were 86.6 ± 6.3%, 84.0 ± 11.1%, 79.3 ± 9.4% and 82.3 ± 7.1%, respectively.

However, the approaches previously reported did not present a good performance to score correctly the microstructure without a posteriori technician revision. Moreover, good results were only obtained for the distinction between A-phases and B-phases, and not for the multi-class discrimination between all the different subtypes. Another drawback was the consideration of simple accuracy that lead to an overestimation of the performance in imbalanced datasets, which is the case of A-phase scoring.

Here, we describe a knowledge discovery methodology in data (KDD) that is for the first time applied to CAP scoring. The KDD encompasses: the extraction of multiple EEG features, different pre-processing options, and different pattern recognition techniques. The KDD aims to inspect about proper processing alternatives for the binary distinction between A-phases and B-phases, and also for multi-class classification of the different CAP components, i.e. the three A-phase subtypes as well as the B-phases. The fully discrimination of the three different A-phases subtypes is a new perspective, since past works tried multi-class approaches but based on grouping of different sub-types, as for example A2 and A3. We also considered the weighted accuracy, in addition to simple accuracy, resulting in a more trustworthy performance assessment. The next section describes the methods used, the database and data characteristics. “Results” section describes the classification performance achieved for the different options considered. Insights about results and future directions are given in “Discussion” and “Conclusion” sections.

## Methods

### Database

The study was carried out on 30 subjects with nocturnal frontal lobe epilepsy, 14 females and 16 males, with ages between 14 and 67 years old (mean = 31.03 ± 11.64). The dataset is available from the *CAP Sleep Database* [18], that comprises several one-night polysomnographic recordings from different patients with different pathologies, and has been used in several studies in the past [10, 11, 13, 17, 19]. The recordings were acquired at the Sleep Disorders Center of the Ospedale Maggiore of Parma, Italy. The polysomnographic data includes at least three EEG channels (F3 or F4, C3 or C4 and O1 or O2, referred to electrodes placed in the earlobes, labeled as A1 or A2), two EOG channels, three electromyography signals (EMG), respiration signals and the ECG. The sampling rates for the recordings vary from 128 to 512 Hz, depending on the patient.

The macrostructure and microstructure scoring were annotated by neurophysiologists and supplied together with the raw EEG data. The macrostructure was annotated according to the R&K rules [20], while CAP was detected in agreement with Terzano reference atlas [7].

### Knowledge detection methodology in data

**Fig. 1:**
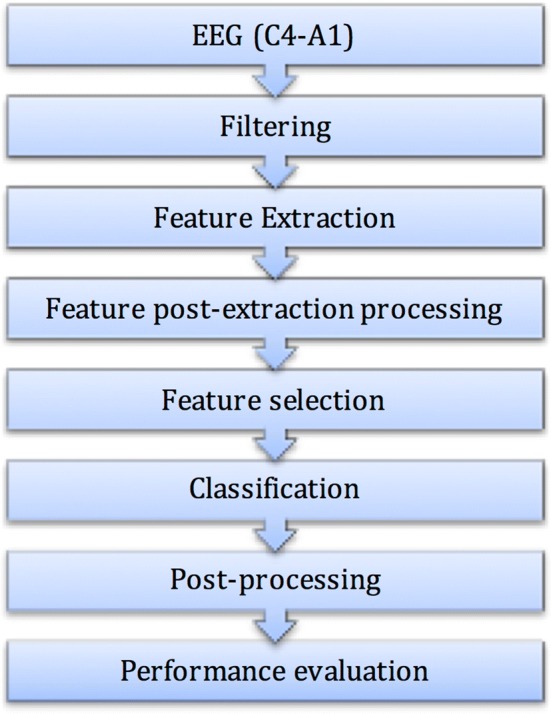
KDD steps for automatic A-phase detection

The main KDD steps are represented in Fig. 1, and they are based in the processing of the monopolar channel C4–A1. In the next sections a closer look into the each one of the steps will be presented.

### Filtering

The original signal was filtered to obtain its components in six frequency bands, i.e. in the band 0.3–35 Hz [originating a signal called in this work as the broadband signal (BB)], and in the five conventional frequency sub-bands. The filter of the signal between 0.3 and 35 Hz was chosen to be in agreement with the usual practice in clinics [21]. The five conventional frequency bands considered were: delta (δ: 0.3–4 Hz), theta (*θ*: 4–8 Hz), alpha (α: 8–13 Hz), sigma (σ: 13–16 Hz) and beta (*β*: 16–35 Hz), that will be designated by conventional frequency bands [8] along this article.

The EEG signal was filtered using a third-order band-pass *Butterworth* filter [22] and was chosen due to its flat and without ripples frequency response in both pass- and stop-bands.

### Feature extraction

**Fig. 2:**
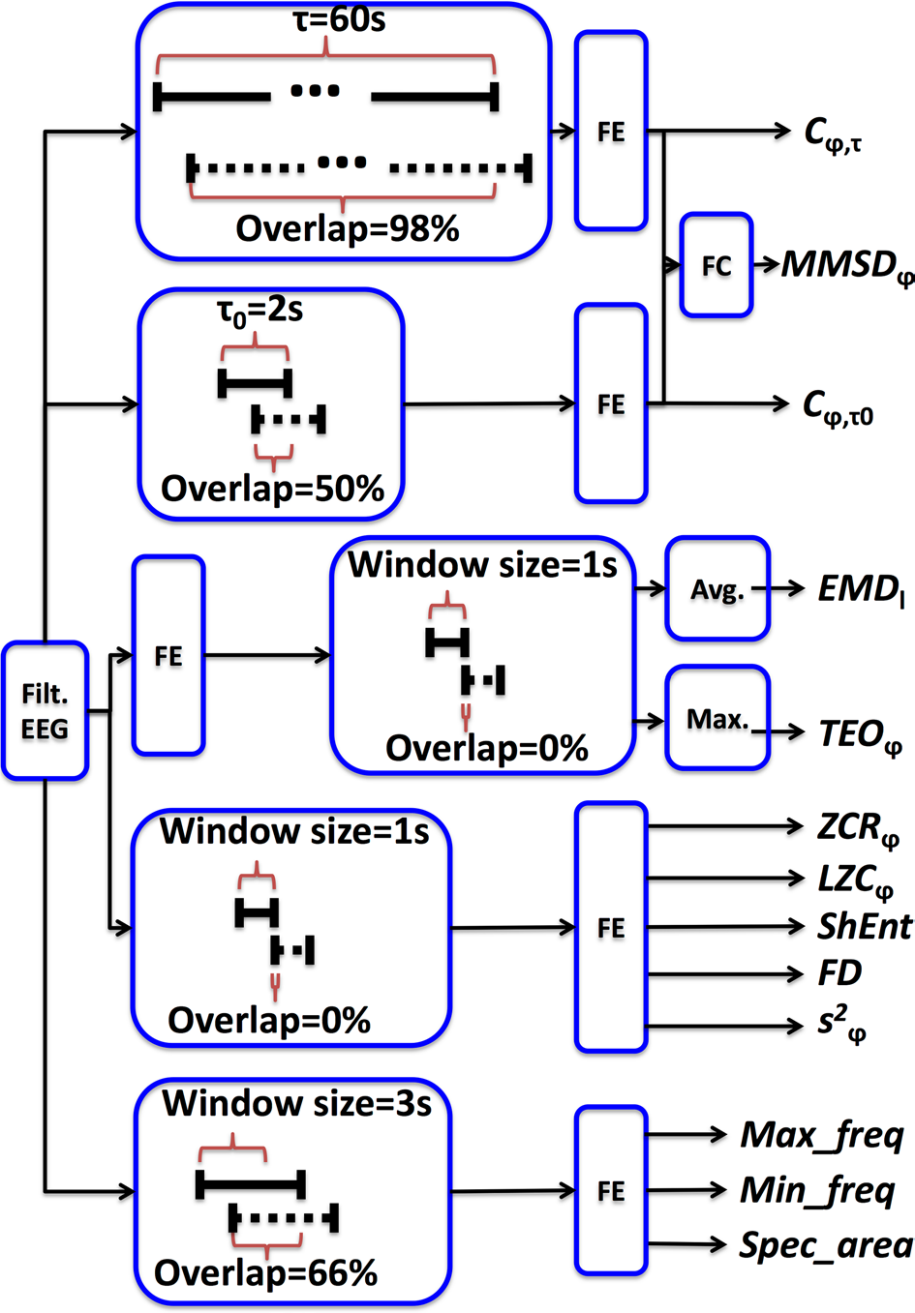
Schematization of the feature computation process based on the filtered EEG signal. In the boxes representing the segmentation process, aiming to represent the percentage of overlap, full lines describe a given window at discrete time k, and dashed lines represent the same window but at time k + 1. “FE” boxes represent feature extraction, and the “FC” box represent the computation of the $MMSD_{\varphi }$ feature that result from the combination of $C_{\varphi , \tau }$ and $C_{{\varphi ,\tau_{0} }}$

Features were extracted from EEG recordings that have a duration of a whole night of sleep (around 8 h). Each feature had a value for each second by segmenting using a sliding window approach. Overall, different window sizes and different degrees of superposition between consecutive windows were considered. The options for the different features are presented in Fig. 2. Also, for two features, segmentation was performed after features computation. The need to define different segmentation procedures was due to the particularities of the different features. Details on the computation of each feature will be described in the following sections.

#### Macro–micro structure descriptor

Macro–micro structure descriptor (MMSD) is an a-dimensional and normalized measurement of how the mean amplitude, *C*, of the EEG signal in a given frequency band $\varphi (\varphi \subset \{ \delta ,\theta ,\alpha ,\sigma ,\beta \} )$) differs, at a given time instant, from its background. The MMSD is given by [1],

1 $$MMSD_{\varphi } = \frac{{C_{{\varphi ,\tau_{0} }} - C_{\varphi ,\tau } }}{{C_{\varphi ,\tau } }}.$$

This means that ${\text{MMSD}}_{\varphi }$ results from the combination of two primary features $C_{\varphi ,\tau }$ and $C_{{\varphi ,\tau_{0} }}$ that were also considered in this work. In a general way, $C_{\varphi ,\tau }$ represents the mean amplitude at a given time instant, computed taking in account the past samples over an interval of size $\tau$. If $\tau$ is long enough, it represents the background of the signal, while if it is too short it is related with instantaneous signal activity. It was reported that if $\tau = 60\;s$ and $\tau_{0} = 2\;s$, $C_{\varphi ,\tau }$ and $C_{{\varphi ,\tau_{0} }}$ are generally related to the sleep macro- and microstructure, respectively [9]. Therefore, these were the values used to compute ${\text{MMSD}}_{\varphi }$, $C_{{\varphi ,\tau_{0} }}$ and $C_{\varphi ,\tau }$. Given the ${\text{MMSD}}_{\varphi }$ dependence on the sleep microstructure, it has been used to classify A-phases [9–11].

One the one hand, given the selected $\tau$ value, ${\text{MMSD}}_{\varphi }$ and $C_{\varphi ,\tau }$ were computed by considering a window of 60 s and a superposition between consecutive windows of 59 s (98% overlap). On the other hand, $\tau_{0}$ defines that $C_{{\varphi ,\tau_{0} }}$ was computed based on a window of 2 s and a superposition between consecutive windows of 1 s (50% of overlap).

#### Teager energy operator

The Teager energy operator (TEO) gives a non-linear measure of the instantaneous signal energy [23, 24]. In its discrete form TEO is given by [2],

2 $${\text{TEO}}\left[ {x[n]} \right] = x[n]^{2} - x\left[ {n - 1\left] {\; \times \;x} \right[n + 1} \right],$$

where *x*[*n*] is the discrete signal sequence. This operator has been successfully used in several signal processing applications. For example, it was used in speech processing [25, 26], and feature extraction from EMG signals [27]. TEO has been referred as adequate for the identification of some EEG elements which are crucial for the CAP scoring, like sleep-spindles [28], and K-complexes [29]. TEO was computed for all the EEG signal and for all frequency bands and was applied in first place to the entire EEG time series due to its non-causal formulation. Afterwards, the TEO sequence was divided into epochs of 1 s and each one is represented by the maximum TEO value.

#### Zero-crossing rate

Zero-crossing rate (ZCR) is a measure of the dominant frequency of a signal, and is obtained in the time domain by counting the number of baseline crossings in a fixed time interval [30]. The ZCR is a fast, intuitive and low complexity way to obtain information about the signal frequency in a short period of time [31, 32]. This feature has been widely used in different applications [33], in particular in the sleep staging field [30, 34]. To compute this feature a non-overlapping moving window of 1 s duration was considered.

#### Lempel–Ziv complexity

Lempel–Ziv complexity (LZC) is a metric used to evaluate the randomness of finite sequences proposed by Lempel and Ziv, in 1976 [35], and has been used to characterize sleep [36, 37]. To compute the LZC complexity a numerical sequence has to be transformed into a symbolic sequence. A frequent approach is to convert the signal, *x*[*n*], into a binary sequence, *P *= *s*[*n*], and comparing the signal with a threshold, *T*_*d*_. The points whose value is greater than *T*_*d*_ are converted to 1, otherwise to 0. Afterwards, a dictionary is build based on the sequences present in the signal *s*[*n*]. The size of the dictionary is proportional to the LZC. The methodology used to compute this feature is described in [38]. LZC as well as ZCR were computed for the six signals: broadband signal and five frequency bands, and using a non-overlapping moving window of 1 s.

#### Discrete time short time Fourier transform

Discrete Fourier transform (DFT) is the simplest form of time–frequency analysis for discrete stationary signals, and is given by [3],

3 $$X\left[ k \right] = \mathop \sum \limits_{n = 0}^{N - 1} x\left[ n \right]e^{{ - j\frac{2\pi kn}{N}}} ,$$

where *N* is the total the number of samples in a given signal segment, *x*[*n*] is the value of the signal at instant *n* and *k* is the discrete frequency bin (0 to N − 1).

The EEG is a non-stationary signal, with different intervals of time having different frequency contents. If the DFT is applied to the signal the evolution of frequencies with time will be lost. To overcome this, the solution is to divide the signal into pieces through the application of a window, *w*, and apply the DFT [39] to each one, leading to the discrete time short time Fourier transform (DTSTFT), given by [4],

4 $$X\left[ {n,k} \right] = \mathop \sum \limits_{m = - \infty }^{\infty } x\left[ m \right]w\left[ {n - m} \right]e^{{ - j\frac{2\pi km}{N}}}$$

The window *w*[*n*] is assumed to be non-zero only in an interval of length *N*_*w*_ and is referred to as the analysis window. The sequence *x*[*m*]*w*[*n *− *m*] is called short section of *x*[*m*] at time *n* [40]. In this work, for each window, it was obtained a spectrum and from it was extracted the frequency of maximum energy ($Max\_freq$), the frequency of mean energy ($Mean\_freq$) and the area under the magnitude spectrum curve ($Spec\_area$). The spectrum was computed considering a window length of 3 s, with an overlap between windows of 2 s.

#### Empirical mode decomposition

In empirical mode decomposition (EMD) a given signal is decomposed in intrinsic mode functions (IMF), where each one represents an embedded characteristic oscillation on a separated time-scale. The EMD application requires a continuous signal, with the number of maxima equal to the number of minima, and also a signal with zero mean. The EMD has been used in EEG applications, for example, for classification of mental tasks [23] and in automatic sleep staging using ECG [41].

In this work EMD was computed for 12 decomposition levels, and the procedure used is available in [42]. To overcome problems with EEG segmentation EMD was applied to the entire signal and segmented afterwards by using consecutive windows of 1 s without overlap. The features derived from EMD were the average values of the different IMFs obtained for each window.

#### Shannon entropy

Shannon entropy (ShEnt) evaluates the randomness (complexity) of a signal by computing its amplitude distribution and the probability for a given value to occur. The higher the probability for the values to happen, the less information exists on the signal, resulting in a smaller entropy. ShEnt has been widely used in EEG processing, for example to distinguish between normal and epileptic EEG [43]. It was proved that this feature is proportional to the sleep macro and microstructure [44] and it has been applied in the automatic sleep staging [45–48]. A non-overlapping moving window of 1 s duration was applied to the broadband signal, then the ShEnt was computed.

#### Fractal dimension

Fractal dimension (FD) quantifies the number of times the same sequence appears in a signal. A signal can be composed by basic building-blocks forming a pattern, the FD quantifies the number of these basic building-blocks. The algorithm proposed by Higuchi [49] is generally used for finding FD in EEG signals.

Fractal dimension is related with sleep macro and microstructure [44] and has been used in the automatic staging of sleep patterns [45, 47]. The broadband signal was divided into epochs of 1 s length, afterwards the FD was computed for each one of them.

#### Variance

Variance has been used for automatic sleep staging [47] and A-phases detection [17]. The formula for variance computation, *s*^2^, is on Equation [5], where *N* is the number of signal samples, $x\left[ n \right]$ is the value of the *n*-th signal sample and $\bar{x}$ the mean value of the signal over the time interval $\left[ {1,N} \right]$.

5 $$s^{2} = \frac{1}{N - 1}\mathop \sum \limits_{n = 1}^{N} (x\left[ n \right] - \bar{x})^{2}$$

Variance was computed by segmenting the broadband EEG signal with a non-overlapping moving window of 1 s duration.

#### Summary

Given the different decomposition levels, and the considered frequency bands, a total of 55 features were obtained. Table 1 summarizes which features were considered and for which frequency band were extracted. At the end, a feature name is composed by its name concatenated with the frequency band considered. For example, the Teager energy extracted for the EEG signal in the $\theta$ band is described by ${\text{TEO}}_{\theta }$. EMD was computed for 12 decomposition levels and is represented by $EMD_{l}$, where $l = 1,2, \ldots ,12$, represents each level. Details about the window size used and percentage of superposition between windows are given in Fig. 2. For ${\text{TEO}}_{\varphi }$ and for $EMD_{l}$ segmentation was performed after feature computation in order to comply with the ${\text{TEO}}_{\varphi }$ non-causal characteristic, and to discard the windowing effect that has drastic consequences for $EMD_{l}$. To mention that the window lengths and overlaps defined guaranties the sampling rate of 1 s for all the features.

**Table 1:** Computed EEG features

Measure	Acronym	Frequency band (*φ*)					
		BB	$\delta$	$\theta$	$\alpha$	$\sigma$	β
Macro–micro structure descriptor	MMSD_*φ*_		✓	✓	✓	✓	✓
	$C_{\varphi ,\tau }$		✓	✓	✓	✓	✓
	$C_{{\varphi ,\tau_{0} }}$		✓	✓	✓	✓	✓
Teager energy operator	${\text{TEO}}_{\varphi }$		✓	✓	✓	✓	✓
Zero-crossing ratio	${\text{ZCR}}_{\varphi }$	✓	✓	✓	✓	✓	✓
Lempel–Ziv complexity	${\text{LZC}}_{\varphi }$	✓	✓	✓	✓	✓	✓
Discrete time	$Max\_freq$	✓					
Short-time	$Mean\_freq$	✓					
Fourier transform	$Spec\_area$	✓					
Empirical mode decomposition	${\text{EMD}}_{l}$	✓					
Shannon entropy	ShEnt	✓					
Fractal dimension	FD	✓					
Variance	$s_{\varphi }^{2}$	✓	✓	✓	✓	✓	✓

BB stands for broadband EEG signal, and $\delta$, $\theta$, $\alpha$, $\sigma$ and $\beta$ are the conventional frequency bands. The $\varphi$ specifies the frequency band considered, and the (✓) symbol indicate if a given measure was extracted for a given frequency band. Concerning $EMD_{l}$, $l$ represents the decomposition level that in this paper can assume valued from 1 to 12

### Features post-extraction processing

Firstly, all features except MMSD, TEO and EMD were smoothed using a causal moving average FIR filter of order 30  [50]. These features were excluded because they detect changes in amplitude and frequency, and if the smoothing technique had been applied, important information could had been lost.

Secondly, the samples four standard deviations away from the mean were considered outliers. Their values were replaced by the median of the feature. If the frequently used value of three standard deviations would be used, the A-phases would likely be considered as outliers. Therefore, the maximum distance allowed was extended. Finally, the values of each feature were normalized to be in the [0–1] range.

### Feature ranking and transformation

The selection or reduction of the features used for the classification task is essential for a good performance of the classifier, on the one hand it is desired that the features should be correlated with the class labels, on the other hand they should not be redundant among themselves. Feature selection and transformation also contributes to reduce the computational cost associated with high dimensionality problems (the “curse of dimensionality”). So, the features must be analyzed aiming to select the best set to use in the classification step. In this work two approaches were used, one for feature selection by ranking and other for feature transformation by projection:

- Minimum redundancy maximum relevance (mRMR): This algorithm ranks the features based on two objectives: obtain the highest correlation between the selected features and class labels (maximum relevance) and reduce the redundancy between features (minimum redundancy). This technique is described in detail in references [51].
- Principal component analysis (PCA): PCA finds the directions in a *d*-dimensional features space where data presents the higher variance, guarantying that these directions are orthogonal among them. The final step, the reduction phase, encompass the selection of some of the directions and the projection of data according to them. The directions are found by computing the eigenvectors of the data covariance matrix [52].

### Classification

Different linear and non-linear classification methods were used in this work aiming to see for which methods and conditions a better performance was achieved. From the previous steps 55 features were obtained, and each feature had a value and a class label for each second, thus we trained the models to classify the EEG signal at every second. The class labels were computed based on the original annotations provided for the raw EEG data, as described in “Database” subsection. The different classification methods considered are described next.

#### Discriminant analysis

Discriminant analysis (DA) [53] has been used in different areas like in statistics, pattern recognition and machine learning. Considering a feature vector *x* of size $\left( {d \times 1} \right)$, and *c* classes $\omega_{k}$ ($k = 1,2, \ldots ,c$). To find the best discrimination function, *g*, the first step is to define the function type. It can be linear, $g\left( x \right) = wx + w_{0}$ and in this case the classifier performs a linear discriminant analysis (LDA), or quadratic, $g\left( x \right) = w_{0} + \mathop \sum \nolimits_{i = 1}^{d} w_{i} x_{i} + \mathop \sum \nolimits_{i = 1}^{d} \mathop \sum \nolimits_{j = 1}^{d} x_{i} x_{j} w_{ij}$, and in this case a quadratic discriminant analysis (QDA) is implemented, where *w* is the weight vector and $w_{0}$ is the bias.

The weights (*w*, $w_{0}$) are computed by minimizing a cost function of the classification errors by using *N* examples as training data:

6 $$J\left( w \right) = \frac{1}{N}\mathop \sum \limits_{n = 1}^{N} \left( {t_{n} - g\left( {x_{n} ,w} \right)} \right)^{2} ,$$

where *t*_*n*_ is the n-th sample of the target vector, i.e. the label of sample $x_{n}$.

In a multi-class problem with *c* classes, where $c > 2$, the discrimination procedure is to compute the *c* discriminant functions. The linear discriminant functions are given by $g_{k} \left( x \right) = w_{k}^{T} x + w_{k,0}$. To each point assign a class $c_{k}$ if $g_{k} \left( x \right)$ assume the highest value among all the discriminant functions.

#### k-Nearest neighbors (k-NN)

k-Nearest neighbors is a non-parametric method, which means that there is no assumption about the underlying pattern distributions. Unlikely the DA the k-NN does not find the best function to divide the space into regions. Instead, the training data is stored in a matrix containing the features and the labels assigned to each pattern. To label a new point, *x*, it is compared with the k-closest training points. The class assigned to *x* is the most prevalent class in these k-points [54].

#### Support vector machines

In its native formulation support vector machine (SVM) finds a decision hyperplane that maximizes the margin that separates the two different classes [55].

Considering the training data $\left\{ {x_{i} ,y_{i} } \right\}$, $i = 1, \ldots ,N$, $t_{i} \in \left\{ { - 1,1} \right\}$, $x_{i} \in R^{d}$, where $t_{i}$ are the class labels and $x_{i}$ the features values. Considering that the data is non-linearly separable, the function which define the hyperplane is [7],

7 $$t_{i} \left( {{\mathbf{w}}^{T} {\mathbf{x}}_{i} + b} \right) \ge 1 - \xi_{i} \quad t \in - 1;1$$

where ${\mathbf{w}}$ is the vector normal to the hyperplane, $\frac{b}{{\parallel {\mathbf{w}}\parallel }}$ is the hyperplane offset to the origin and $\xi$ is a quantification of the degree of misclassification. ${\mathbf{w}}$ and *b* are obtained by minimizing [8],

8 $$\begin{array}{*{20}r} \hfill {\varPsi \left( w \right) = \frac{1}{2}\parallel {\mathbf{w}}\parallel^{2} + C\mathop \sum \limits_{i = 1}^{N} \xi_{i} ,\quad {\text{subject to}}} \\ \hfill {y_{i} \left( {{\mathbf{w}}^{T} {\mathbf{x}}_{i} + b} \right) \ge 1 - \xi_{i} ,\quad i = 1,..,N,} \\ \end{array}$$

due *C* defines the influence of $\xi$ on the minimization criterion $\varPsi$.

In the previous case, the boundary that separates the classes is linear. To improve classification in non-linearly separable data the features space is projected into a higher dimensional space based on a Kernel projection where the linear separability principle can be applied. The most popular Kernel, which is used in this work, is the Gaussian Kernel [9],

9 $$k\left( {x_{i} ,x_{j} } \right) = e^{{\gamma \parallel x_{i} - x_{j} \parallel^{2} }} ,\quad \gamma = \frac{1}{{2\sigma^{2} }},$$

where $x_{j}$ is the position of the Gaussian centre and $\sigma$ the Gaussian aperture (standard deviation).

The SVM is defined as a two-class classifier, but A-phase classification is a multi-class problem. The usual approach for multi-class SVM classification is to use a combination of several binary SVM classifiers. Different methods exist but in this work we used the one-against-all multi-class approach [56]. This method transforms the multi-class problem, with *c* classes, into a series of *c* binary sub problems that can be solved by the binary SVM. The *i* th classifier output function $\rho_{i}$ is trained taking the examples from $c_{i}$ as 1 and the examples from all other classes as − 1. For a new example *x*, this method assigns *x* to the class associated with the largest value of $\rho_{i}$ [56].

### Post-processing

The unique post-processing implemented was focused on the validation the A-phase duration that must be within the interval 2 s to 60 s. Thus, the number of consecutive 1 s epochs classified as A-phases were quantified. If a single epoch was classified as A-phase, or if more than 60 consecutive epochs were classified as A-phases, they were considered as background signal, i.e. as B-phases.

### Performance evaluation

The Leave-one-out approach [57] was used, which is the most classical exhaustive cross validation (CV) procedure. Supposing that the data is composed by *M* patients, the algorithm will be trained *M* times. In each iteration, a different patient is “left out” to be the test data and the remaining ones are training data. The algorithm output for each patient is compared to the real staging by filling confusion matrices. We evaluated the algorithms from two perspectives: the performance on detecting A-phases from B-phases (binary problem) and the algorithms capabilities to differentiate among the different CAP subcomponents, i.e. the capability to differentiate A1, A2, A3 and B phases (multi-class problem). Thus, two confusion matrices were filled, which can be represented by the generic confusion matrix in Table 2.

**Table 2:** Generic confusion matrix, where “^” indicates predictions provided by the algorithms

Predicted	True			
	$\varvec{c}_{1}$	$\varvec{c}_{2}$	$\cdots$	$\varvec{c}_{\varvec{K}}$
$\widehat{\varvec{c}}_{1}$	$n_{1,1}$	$n_{1,2}$	$\cdots$	$n_{1,K}$
$\widehat{\varvec{c}}_{2}$	$n_{2,1}$	$n_{2,2}$	$\cdots$	$n_{2,K}$
$\vdots$	$\cdots$	$\cdots$	$\cdots$	$\cdots$
$\widehat{\varvec{c}}_{\varvec{K}}$	$n_{K,1}$	$n_{K,2}$	$\cdots$	$n_{K,K}$

For the binary problem *c* = {A-phase, B-phase}, while for the multi-class problem *c* = {B-phase, A1, A2, A3}. In Table 2 the diagonal terms $n_{ij}$, where $i = j$, correspond to the instances where the algorithm’s output was consistent with to the real class label, i.e. the true positive patterns. The values $n_{ij}$, where $i \ne j$, are the number of instances misclassified by the algorithm, which can be considered as false positives or false negatives depending on the class under analysis.

Considering a given class *k*, four measurements were taken:

- True positive ($TP_{k}$): number of instances correctly classified as class *k*;
- False positive ($FP_{k}$): number of instances classified as *k* when in fact they belong to other class;
- True negative ($TN_{k}$): number of instances correctly not classified as *k*;
- False negatives ($FN_{k}$): number of instances assigned to other classes when in fact they belong to class *k*;

For a classification problem with *K* classes, these measurements can be computed from the confusion matrix as follows in [10].

10 $$\begin{aligned} n_{ + ,k} & = \mathop \sum \limits_{i = 1}^{K} n_{i,k} \\ n_{k, + } &= \mathop \sum \limits_{j = 1}^{K} n_{k,j} \\ TP_{k} & = n_{k,k} ; \hfill \\ FP_{k} & = n_{k, + } - n_{k,k} ; \\ FN_{k} &= n_{ + ,k} - n_{k,k} ; \\ TN_{k} &= N - TP_{k} - FP_{k} - FN_{k} \\ \end{aligned}$$

To evaluate the algorithm’s three performance measures were used: sensitivity (SE), specificity (SP) and accuracy (AC), which are given by [11]:

11 $$\begin{aligned} & SE_{k} = \frac{{TP_{k} }}{{TP_{k} + FN_{k} }};\quad SP_{k} = \frac{{TN_{k} }}{{TN_{k} + FP_{k} }}; \hfill \\ &AC = \frac{{TN_{k} + TP_{k} }}{{TN_{k} + FP_{k} + FN_{k} + TP_{k} }}. \hfill \\ \end{aligned}$$

The B-phases, i.e. background epochs, were more present than the A-phases, sometimes seven times more. Therefore, when an algorithm correctly detected most of the B-phases, even though performance was not as good as A-phases, it usually had a higher accuracy. Since the main objective was to correctly detect the A-phases, the AC might not yield a correct view of the overall performance of the algorithm. A more adequate performance measure was considered, the weighted accuracy (WAC), that takes into account the number of instances of each class. Thus, a miscomputation of B-phase instance will have lower impact on accuracy than an A-phase misclassification. The weighted accuracy is given by [12].

12 $${\text{WAC}} = \frac{1}{K}\mathop \sum \limits_{j = 1}^{K} \frac{{n_{jj} }}{{n_{ + ,j} }}.$$

AC is also presented, although it provides poorer performance, to better compare the results with those reported in literature.

## Results

In total, 55 features were computed. An evaluation of the performance using different number of features and principal components to build a classifier was performed and the results are shown in the following sub-sections. A straightforward approach was implemented and worked as follow: (i) at start only the two more important features or principal components were considered; (ii) then we introduced the next feature/principal component in the next step; (iii) until all the features/components were considered as classifier’s input.

### Multi-class A-phase classification

#### mRMR

Features ranking with mRMR did not returned exactly the same result for all the patients, being an evidence of the inter-individual variability. Analyzing all the rankings sequences for each patient, the most prevalent ranking sequence was (from higher to lower importance): ${\text{LZC}}_{BB}$, $C_{\beta ,\tau }$, $C_{\sigma ,\tau }$, $C_{{\beta ,\tau_{0} }}$, $C_{{\sigma ,\tau_{0} }}$, $C_{\delta ,\tau }$, ${\text{ZCR}}_{BB}$, $C_{{\delta ,\tau_{0} }}$, $Max\_freq$, $C_{{\alpha ,\tau_{0} }}$, $C_{\theta ,\tau }$, ${\text{MMSD}}_{{}}$, $C_{{\theta ,\tau_{0} }}$, $C_{\alpha ,\tau }$, ${\text{TEO}}_{\delta }$, ${\text{MMSD}}_{{}}$, ${\text{EMD}}_{1}$, $s_{\delta }^{2}$, ${\text{MMSD}}_{\sigma }$, ${\text{EMD}}_{2}$, ${\text{EMD}}_{6}$, ${\text{MMSD}}_{\alpha }$, ${\text{EMD}}_{5}$, ${\text{EMD}}_{7}$, ${\text{MMSD}}_{\beta }$, $s_{\theta }^{2}$, ${\text{MMSD}}_{\sigma }$, $Spec\_area$, $s_{BB}^{2}$, ${\text{EMD}}_{10}$, ${\text{EMD}}_{4}$, $s_{\beta }^{2}$, ${\text{EMD}}_{11}$, ${\text{EMD}}_{12}$, ShEnt, ${\text{EMD}}_{3}$, $Mean\_freq$, $s_{\sigma }^{2}$, ${\text{TEO}}_{\theta }$, ${\text{EMD}}_{9}$, ${\text{EMD}}_{8}$, $s_{\alpha }^{2}$, ${\text{TEO}}_{\beta }$, ${\text{TEO}}_{\sigma }$, ${\text{TEO}}_{\alpha }$, ${\text{ZCR}}_{\delta }$, ${\text{ZCR}}_{\theta }$, ${\text{ZCR}}_{\alpha }$, ${\text{ZCR}}_{\sigma }$, ${\text{ZCR}}_{\beta }$, ${\text{LZC}}_{\delta }$, ${\text{LZC}}_{\theta }$, ${\text{LZC}}_{\alpha }$, ${\text{LZC}}_{\sigma }$, ${\text{LZC}}_{\beta }$ and FD.

**Fig. 3:**
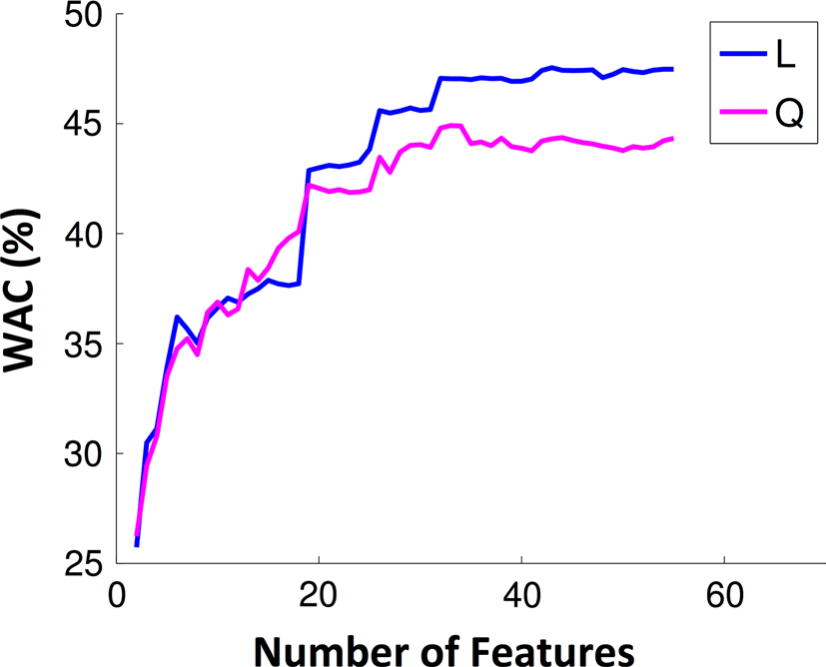
Evolution of WAC with the number of features used to build a model with LDA (L) and QDA (Q)

Using LDA and QDA the performance monotonically increases with dimensionality and can be considered stable for more than 30 features, as presented in Fig. 3. The LDA classifier achieved in general better results than QDA, and when 30 features were considered, the LDA classifier achieved 47% and 61% of WAC and AC, respectively.

**Fig. 4:**
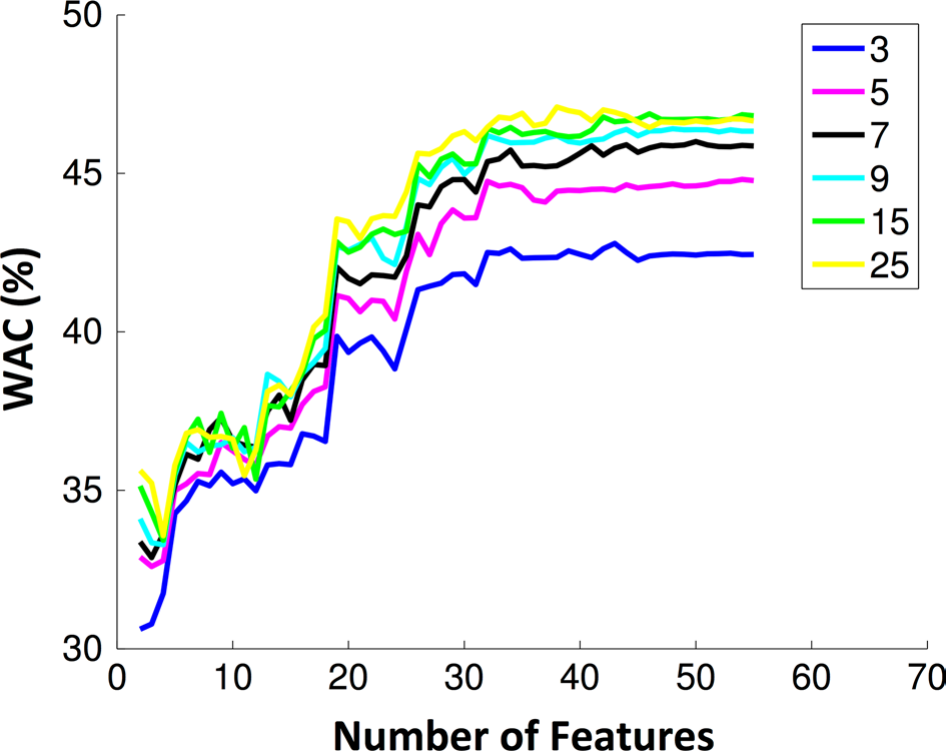
Weighted accuracy evolution with the number of features using a k-NN classifier for different k-values [3, 5, 7, 9, 15, 25]

**Fig. 5:**
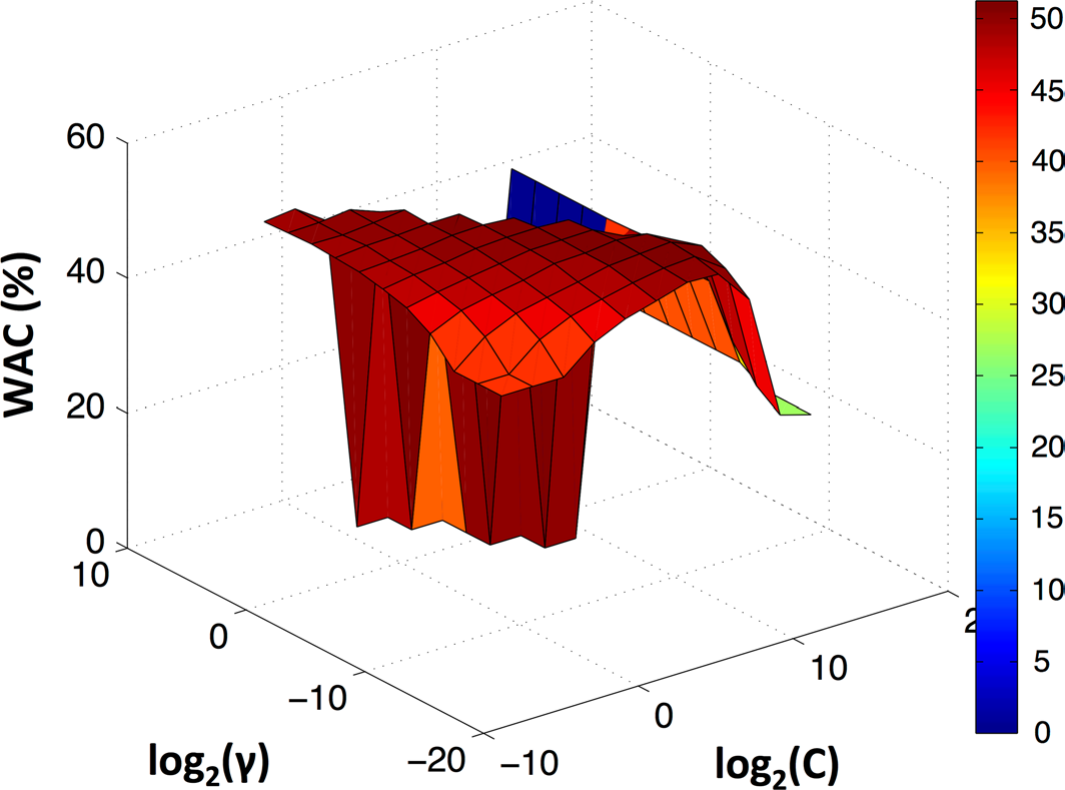
Weighted accuracy for different SVM parameters and considering 40 features (chosen by mRMR). The grid search was performed with $C = 2^{ - 5} ,2^{ - 3} , \ldots ,2^{15}$ and $\gamma = 2^{ - 15} ,2^{ - 13} , \ldots ,2^{5}$

For the k-NN classifier different values of *k* [3, 5, 7, 9, 15, 25] were used to evaluate the performance. The behaviour of accuracy with the number of features is similar to the DA classifier. For the same number of features, the accuracy was similar independently of the *k* value. However, WAC clearly depended on the number of neighbours, and the best *k* that achieved higher weighted accuracy was 25, as depicted in Fig. 4. Considering a k-NN model with 30 features the mean values for WAC and AC were 46% and 70%, respectively. In both classifiers (DA or k-NN) the AC and WAC values stabilized after 30 features, independently of the classifier parameters. Since the computational cost increases with the number of dimensions used, the SVM models were build using only 40 features, which is a number that guaranties a good accuracy when DA and k-NN were applied. A grid search was performed for $C = 2^{ - 5} ,2^{ - 3} , \ldots ,2^{15}$ and $\gamma = 2^{ - 15} ,2^{ - 13} , \ldots ,2^{5}$ as presented in Fig. 5. With this classifier, the best performance was obtained for $C = 2^{ - 1}$ and $\gamma = 2^{ - 1}$, being 51% and 71% the mean WAC and AC, respectively.

The confusion matrix represented in Table 3, shows that for SVM with $C = 2^{ - 1}$ and $\gamma = 2^{ - 1}$, it can be seen that the B-phases were confused with the A1 and A2 subtypes in average 11% of the times, and confused with A3 subtype only 7% of the times. 22% of the A1 phases were confused with A2, but only 5% with A3. A2 was more confused with A1, although the algorithm considered 21% of these phases as B-phases. A3 was the one with lower sensitivity and its misclassification is quite high: 32% were confused with A2, 27% with B-phase and 17% with A1. The A3 and A1 were the most distinct types, thus it was expectable that they were less confused.

**Table 3:** Mean of confusion matrix for an SVM classifier using the 40 features ranked by mRMR, for the parameters of C = 2^−1^ and γ = 2^−1^

Predicted	True			
	B-Phase	A1	A2	A3
B-Phase	*76 ± 5*	15 ± 8	21 ± 12	27 ± 11
A1	11 ± 4	*59 ± 14*	27 ± 13	17 ± 10
A2	11 ± 6	22 ± 12	*44 ± 15*	32 ± 11
A3	7 ± 2	5 ± 4	8 ± 8	*24 ± 10*

Italic font indicates the matrix main diagonal that represent the correctly predicted instances

#### PCA

**Fig. 6:**
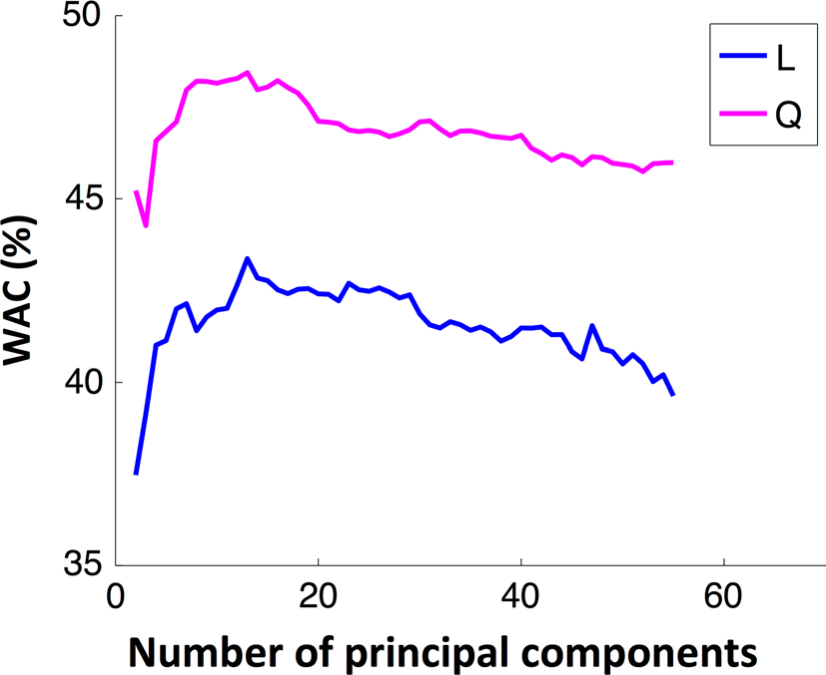
Evolution of WAC with the number of PCA principal components used to build a model with LDA (L) and QDA (Q)

Using a DA classifier QDA achieved greater accuracy values than LDA, for all dimensions. More precisely, accuracy stabilized around 70% for more than 12 dimensions. Although the WAC significantly dropped for a classifier with more than 12 dimensions as can be observed in Fig. 6. A WAC of 49% was achieved using 12 components, that corresponded to an AC of 68%.

**Fig. 7:**
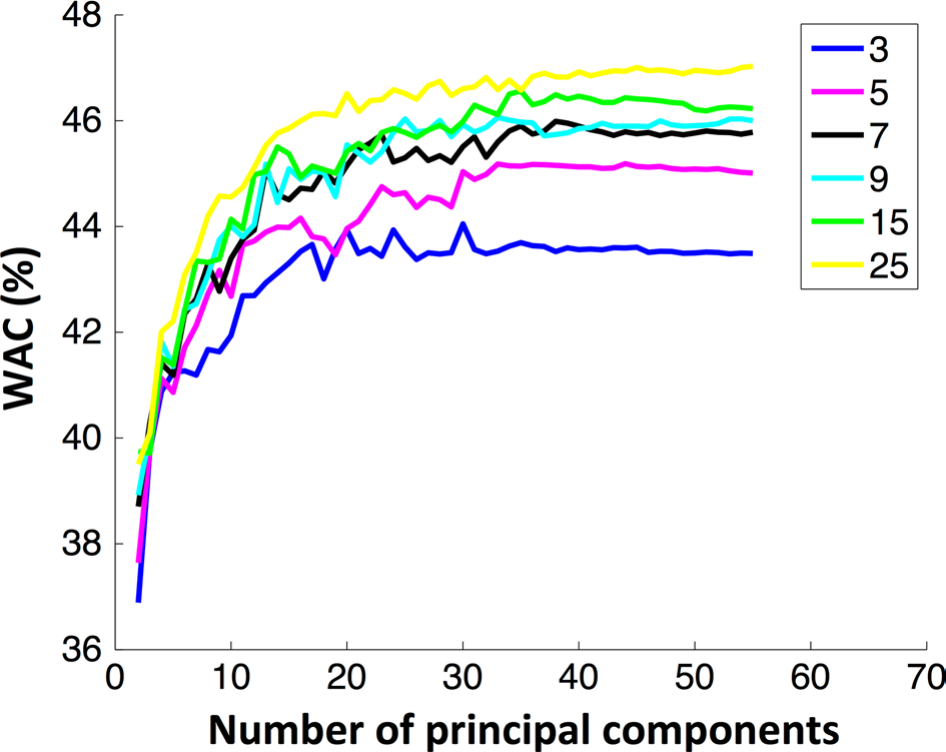
Weighted accuracy evolution with the number of PCA principal components using a k-NN classifier for different k-values [3, 5, 7, 9, 15, 25]

The results obtained with a k-NN classifier using PCA components was quite similar with the ones obtained with feature selection by mRMR, as presented in Fig. 7. Besides this similarity the performance using PCA started to stabilize for more than 30 components as when using mRMR. The WAC registered for 40 principal components and for k = 25 was 47%, corresponding to an AC of 61%.

**Fig. 8:**
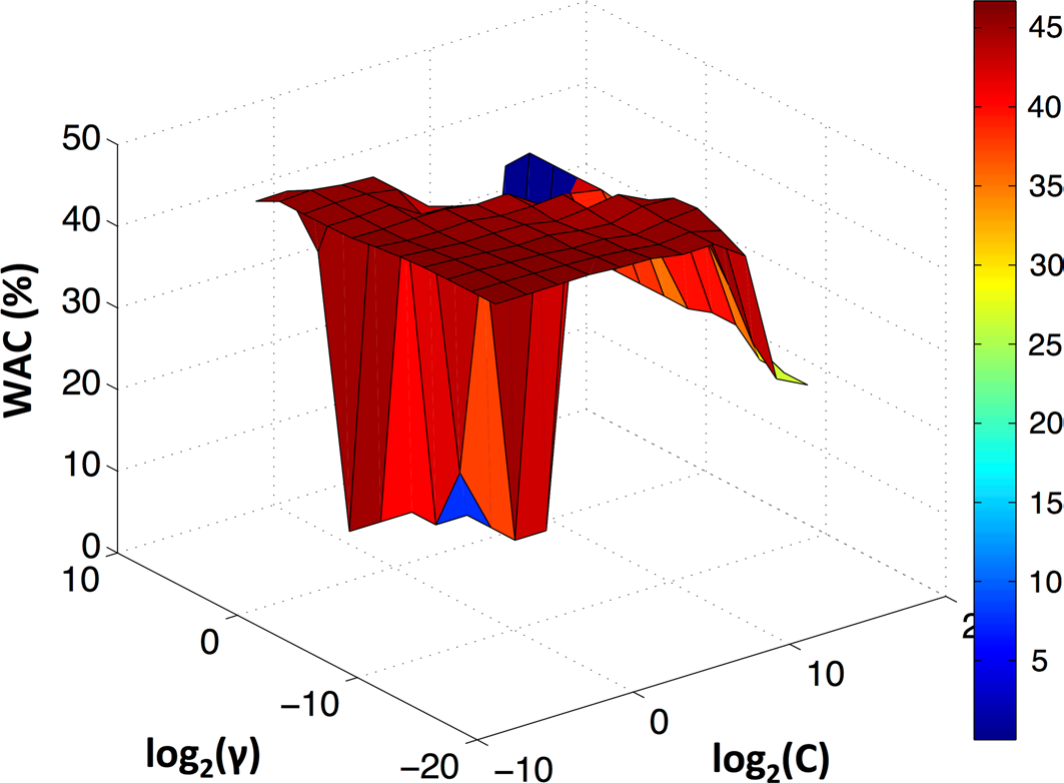
Weighted accuracy for different SVM parameters and considering 30 PCA principal components. The grid search was performed with $C = 2^{ - 5} ,2^{ - 3} , \ldots ,2^{15}$ and $\gamma = 2^{ - 15} ,2^{ - 13} , \ldots ,2^{5}$

Using features selected by mRMR both DA and k-NN started to stabilize for more than 30 dimensions. Although, when PCA was used for a DA classifier WAC achieved a maximum for 12 features and afterwards started to decrease. In k-NN the performance increased until 30 principal components, and for more principal components the performance remained almost constant. Given the previous results of DA and k-NN it was chosen to use a SVM classifier with the first 30 more important principal components. The WAC results obtained after a grid search are presented in Fig. 8. The best SVM parameters were $C = 2^{ - 5}$ and $\gamma = 2^{ - 9}$, resulting in WAC and AC of 47% and 56%, respectively.

The confusion matrix is shown in Table 4. With mRMR feature selection the results are more dispersive than when using PCA, i.e. when a class was misclassified using PCA it was mostly confused with one class rather than with two. An interesting observation for the combination of SVM and PCA was that the majority of A1, 32%, were mistaken with A2 and only 5% and 2% were misclassified as B-phase and A3-phase, respectively. Contrarily, in mRMR 22% of the real A1 were wrongly classified as A2 and 15% confused with B-phases.

**Table 4:** Mean of confusion matrix for a SVM classifier using 30 principal components, for the parameters of C = 2^−5^ and γ = 2^−9^

Predicted	True			
	B-Phase	A1	A2	A3
B-Phase	*61 ± 3*	5 ± 2	9 ± 4	26 ± 9
A1	17 ± 4	*60 ± 17*	35 ± 12	25 ± 8
A2	16 ± 4	32 ± 15	*53 ± 10*	38 ± 9
A3	6 ± 2	2 ± 3	3 ± 3	*13 ± 5*

Italic font indicates the matrix main diagonal that represent the correctly predicted instances

### Binary classification of A-phase vs B-phase

For the two-class problem, where it was only necessary to detect the A-phases from the B-phases without concerning the A-phase subtype, all the classifiers applied to the previous problem are analyzed in the following.

#### mRMR

With DA, the higher value of WAC was 72% with a LDA classifier, and using the 43 best features. The registered SP, SE and AC were: 65%, 78% and 67%, respectively. Using k-NN with $k = 25$ the better performance values were obtained for 55 features, being AC equal to 75% and WAC equal to 80%. In these conditions, sensitivity and specificity were 82% and 68%, respectively. Considering the SVM with 40 features, the highest WAC achieved was 78% for $C = 2^{ - 1}$ and $\gamma = 2^{ - 1}$, assuming AC, SP and SE the values 76%, 79% and 77%, respectively.

#### PCA

Combining the principal components with DA classifier the higher WAC obtained was 76% for a QDA with 15 dimensions. In this case SE, SP and AC were 79%, 74% and 73%, respectively. Regarding k-NN, the highest WAC, 75%, was achieved for 54 components and for $k = 25$. SE, SP and AC were 86%, 65% and 68%, respectively. For SVM the best result was for 40 principal components and for $c = 2^{ - 5}$ and $\gamma = 2^{ - 11}$. The achieved WAC was 75%, being AC, SE and SP: 73%, 76% and 73%, respectively.

### Summary

Table 5 presents an overview of the results obtained with the different classifiers, with feature selection or reduction, and for the binary or multi-class classification problem. One can observe that feature selection by mRMR led to the attainment of the best results in both classification scenarios. For the multi-class problem, the best classifier was SVM, and for the binary case k-NN was the one that attained the better performance.

**Table 5:** Summary of the best results obtained for all the main methodological options considered

	Multi-class problem						Binary problem					
	mRMR			PCA			mRMR			PCA		
	#F.	AC (%)	WAC (%)	#P. C.	AC (%)	WAC (%)	#F.	AC (%)	WAC (%)	#P. C.	AC (%)	WAC (%)
DA	30	61	47	12	68	49	43	67	72	15	73	76
k-NN	30	70	46	40	61	47	55	75	*80*	54	68	75
SVM	40	71	*51*	30	56	47	40	76	78	40	73	75

The best WAC results for each classification scenario (binary or multi-class) are in italic. #F. and #P. C. stand for number of features and number of principal components, respectively

## Discussion

To the best of our knowledge, only two methods that discriminate between A-phases subtypes were found in literature, proposed by Barcaro et al. [11] and by Navona et al. [10]. In these methods, the subtypes A2 and A3 were joined and classified as one, A2/A3. Aiming to improve comparison, the algorithms described in [10, 11] were implemented in this work and their results, as well as the results for the other methods, are compiled in Table 6. It can be seen that the sensitivities of A2/A3 were quite low, in the two versions proposed by Barcaro et al. (below 10%). Although, the AC of this method was high. This value was due to class imbalance, i.e. due to a high predominance of the B-phases, which occupied 70% of the all data. Then, due to a high sensitivity of B-phases, AC will be high as well, even if the sensitivity of A-phases was low. WAC in turn was lower than AC, quantifying the low classification of the A2/A3 class. The KDD implemented for A-phases discrimination enabled the development of classification solutions to detect all the three A-phases subtypes, which is an innovation. All the A-phases sensitivities achieved were higher than the published methods.

**Table 6:** Comparison of the best classifier model proposed in this work with the ones presented in literature, that distinguish among A-phases subtypes

Method	Publication	Specificity	Sensitivity			AC (%)	WAC (%)
		(B-phase)	A1	A2	A3		
DA	This work	73	66	37	18	68	49
k-NN	This work	71	59	31	16	70	46
SVM	This work	76	58	44	24	71	51
MMSD	[10]	89	51	6.5		79	49
	[11]	79	57	10		72	49

Most of the A3 were arousals that are events with characteristics of awake sleep stage. Therefore, the classifiers were trained with B-phases similar to A3 phases, which led to a lot of A3 phases being wrongly classified as B-phase. This can be seen observing the confusion matrices represented in Tables 3, 4. The typical waveform associated with A1 are only the K-complex that counts with appropriate features for its detection, which explained the higher results for this subtype, compared to the A2 and A3.

Subtypes sensitivities can be improved by taking into account context information. Information from the macro-structure could be important to improve A3 discrimination, given that it is confused with the awake state. Other aspect that is important to take into account is the subject condition. For example, NFLE patients, to which belongs the population considered in this work, are characterized by a significant enhancement of all A-phases subtypes when compared with controls [58]. In addition, A-phases offers favorable conditions for the occurrence of nocturnal motor seizures [6], and for the generation of paroxysmal EEG features that can be used as bio-markers.

The majority of the algorithms presented in literature were developed for A-phase vs B-phase detection, as it can be seen in the literature review. The performance of the proposed algorithms with those in the literature are compared in Table 7.

**Table 7:** Comparison of the best classifier model proposed in this work with the ones present in literature to detect A-phases

Method	Publication	SE (%)	SP (%)	AC (%)	WAC (%)
DA	This work	79	74	73	76
	[17]	72	87	85	–
k-NN	This work	82	69	75	80
SVM	This work	79	76	76	78
	[17]	70	84	82	–
MMSD	[9]	58	81	78	71
	[10]	52	89	83	69
	[11]	58	80	78	73

In general, the accuracy reported in literature is higher than the ones reported in this work for the various methods. This is due to the fact that they reported the standard accuracy without accounting for class imbalance. Therefore, again, they gave more importance to the B-phases that were more numerous than the A-phases. The consideration of WAC enabled a more appropriate estimation of the algorithms performance. In fact, if WAC was considered instead of AC, the algorithms presented in [9–11], presented much lower performance values, as can be observed in Table 7.

## Conclusion

We propose a multi-step KDD to determine appropriate processing options for automatic CAP scoring. We extracted several features, approached two different feature selection/reduction methods, and different classification methods. It was concluded that using the features ranked by mRMR without any transformations leads to better results. It was shown that the SVM was the best classification method for the full discrimination of all CAP components, while k-NN performed better when one just wants to discriminate A-phases from B-phases.

The proposed KDD is for the first time applied to CAP scoring. In particular, the fully discrimination of the three different A-phases subtypes is a new perspective, since past works tried multi-class approaches but based on grouping of different sub-types. We also considered the weighted accuracy, in addition to simple accuracy, resulting in a more trustworthy performance assessment. Globally, better subtype sensitivities than other published approaches were achieved.

Future steps to improve classification will encompass the consideration of context information related with CAP classification, as for example sleep stage and subject medical condition. Other future step is the analysis of the benefits of micro-structure staging as a disease biomarker, as for example as a precursor of epileptic seizures.
